# Clinical Predictors of Relapse in a Cohort of Steroid-Treated Patients With Well-Controlled Myasthenia Gravis

**DOI:** 10.3389/fneur.2022.816243

**Published:** 2022-02-04

**Authors:** Shengyao Su, Lin Lei, Zhirong Fan, Shu Zhang, Qi Wen, Jingsi Wang, Yan Lu, Li Di, Min Wang, Hai Chen, Yuwei Da

**Affiliations:** Department of Neurology, Xuanwu Hospital, Capital Medical University, Beijing, China

**Keywords:** myasthenia gravis, relapse, steroid monotherapy, clinical predictor, steroid reduction, steroid withdrawal

## Abstract

**Objective:**

Despite the high efficiency of glucocorticoids (GCs), ~18–34% patients with myasthenia gravis (MG) may experience relapses of the disease. Here, we aim to identify clinical factors related to relapses during steroid tapering or after withdrawal in MG patients who were well-managed on steroid monotherapy.

**Methods:**

We conducted a retrospective study on 125 MG patients from the Xuanwu Hospital MG Trial Database. Patients were treated with corticosteroids and achieved minimal manifestation status (MMS) or better. Patients were divided into steroid reduction subset (*N* = 74) and steroid withdrawal subset (*N* = 51). Clinical characteristics and therapeutic data were compared between patients with disease relapse and those who maintained clinical remission at the last follow-ups. Cox proportional hazards regression models were used to identify risk factors of relapse in each subset.

**Results:**

Thirty-seven (29.6%) patients experienced relapses during the follow-up periods. Relapse during the steroid reduction was significantly associated with drug reducing duration (HR = 0.81, 95%CI 0.74–0.89, *P* < 0.001). Risk of relapse was augmented if the drug reducing duration was <11.5 months (HR 27.80, 95%CI 5.88–131.57, *P* < 0.001). Among patients who discontinued the steroids, those with onset symptoms of bulbar weakness (adjusted HR 3.59, 95%CI 1.19–10.81, *P* = 0.023) were more likely to experience relapse.

**Conclusion:**

Our study demonstrated that patients could benefit from prolonged steroid-reducing duration to prevent disease relapse. Patients with bulbar weakness at disease onset should be proposed to take long-term steroids or other immunosuppressants.

## Introduction

Myasthenia gravis (MG) is an autoimmune disease with the presence of autoantibodies against the neuromuscular junction proteins. Treatments such as acetylcholinesterase inhibitors, immunotherapies, thymectomy, intravenous immunoglobulin (IVIG), and plasma exchange are used to realize the therapeutic target of full physical function and high quality of life (1). Despite the fact that promising novel therapies are upcoming (2), the glucocorticoid (GC) is still the first choice of MG therapy on the basis of rapid onsets of effects, low costs, and high efficiency, which could lead to improvement in 80–95% patients (3–6). After the relief of symptoms, the corticosteroid dose is reduced or even discontinued to minimize the accompanying side effects of long-term use (3–6). However, ~18–34% patients may experience subsequent exacerbations or disease relapses (5, 7, 8). Only 10–20% patients could discontinue immunotherapy completely and achieve complete stable remission (CSR) (4, 9, 10).

It has been demonstrated that the increased risk of relapse was correlative with drug withdrawal or rapid reduction of steroids when patients took corticosteroid as monotherapy in the 1990's (5, 6). Thereafter, the “slow and steady” tapering strategy was adopted in the clinical practice when the steroid was administrated solely (11). Even so, patients may still experience disease recurrence during the drug reduction. In some cases, relapses of MG occur in months to years after the discontinuation of prednisone (6). However, there are few studies concerning the clinical factors that are correlative with relapse during steroid tapering (5, 6, 12). Moreover, to our knowledge, risk factors of relapse after steroid withdrawal have not been investigated thoroughly.

Here, we present a retrospective cohort analysis of GC-treated MG patients from a single center in order to determine indicators of clinical relapse under steroid monotherapy.

## Materials and Methods

### Patients and Ethical Statements

Medical records and follow up data of consecutive MG patients from the Xuanwu Hospital Capital Medical University Myasthenia Gravis Trial Database since April 2017 to July 2020 were retrospectively reviewed and analyzed. The study was approved by the Ethics Committee of Xuanwu Hospital (No. 2017084). All patients provided written informed consents.

**Figure 1:**
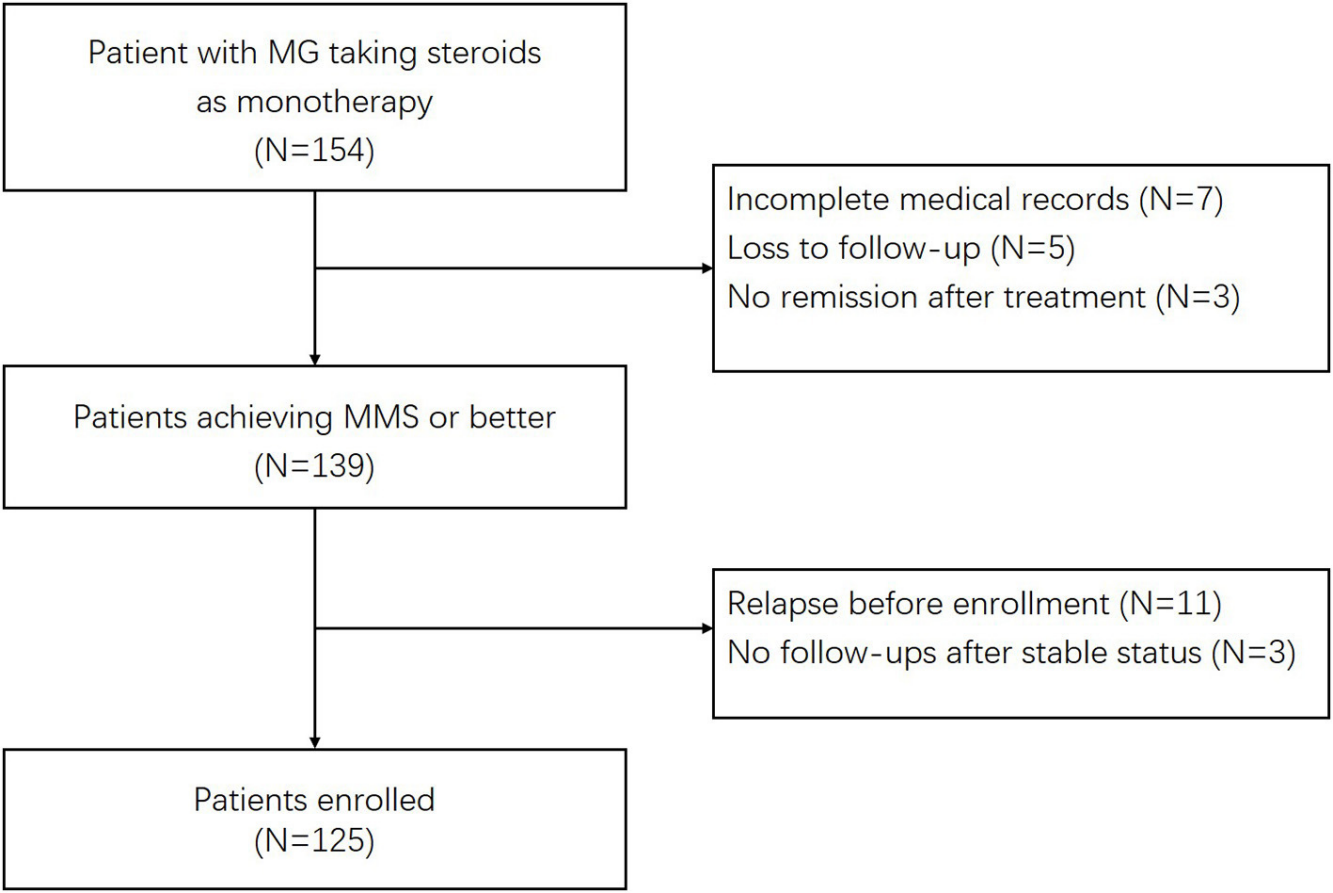
Flowchart of the participants included in the current study.

The inclusion criteria included: (1) Patients were diagnosed with MG and over 16 years. The diagnosis of MG was based on fluctuating weakness symptoms along with supporting pharmacological, serologic, and electrophysiologic tests (13). (2) Patients were treated with GC for controlling disease and the maintenance therapy in the absence of other immunosuppressive agents, except for short-term IVIG during the acute exacerbations. Steroids were prescribed for at least 1 month before patients reached a stable status. The stable status was defined as patients having no symptoms of functional limitations from MG, meeting the criteria for minimal manifestation status (MMS) or better according to the MGFA postintervention status (MGFA-PIS) classification (14). (3) Patients were followed up prospectively after the enrollment for at least 12 months. We identified 154 potential patients who were receiving GC therapies. The exclusion criteria were as follows: (1) patients had incomplete medical records or less than one follow-up visit. (2) Patients took other immunosuppressants, except for short-term IVIG. Patients whose therapies switched to other immunosuppressive agents due to steroid-induced side effects during the follow-up period were excluded. (3) Patients experienced relapses before enrollments. (4) Patients achieved stable status at the last visits with no further follow-up information. Ultimately, 125 patients were enrolled (Figure 1).

### Clinical Features and Evaluations

Clinical features were collected including sex, the age of onset, onset symptoms, symptoms at nadir, MGFA classification at the nadir, presence of autoantibodies, repetitive nerve stimulation tests (RNS) result, and presence of other autoimmune diseases. The presenting symptoms within the first month of disease onsets were collected as the onset symptoms. Mild disease was defined as MGFA II class at disease maximal worsening, and moderate to severe disease was defined as MGFA III to V classes. Radiographic examinations of the mediastinum were performed routinely, and 31 patients underwent thymectomies. Patients with thymoma (*N*=17) were pathologically diagnosed. MG–activities of daily living (ADL) scores were measured to quantify the disease severities. Follow-up assessments were scheduled every 3 months for the first year and then every 6 months. Assessments included clinical symptoms, ADL scores, prednisone doses, and the dates of achieving stable status. Once patients achieved MMS or better, they did not need to come for the return visits and telephone follow-ups would be performed. The follow-ups for all the enrolled patients were performed prospectively and completed by July 2021.

### Treatment

All the enrolled patients were taking steroids as monotherapy. The short-term use of IVIG during the acute exacerbation was permitted. Whether patients were treated with high dose intravenous methylprednisolone (IVMP) pulses or not was also recorded. The induction therapy regimens were categorized as steroid increasing regimen, medium-dose regimen, and steroid-tapering regimen. After patient's marked improvements or reaching MMS or better, the maximal GC doses would be tapered to the minimal doses, if the conditions permitted. The tapering strategy comprised a 5 mg reduction monthly or slower until it reached 20 mg daily, 5 mg reduction every 2 to 4 months until 5 mg daily. A 5 mg dose per day of steroid would be discontinued in 3–6 months. Patients whose GC doses were reduced but not discontinued were grouped into steroid reduction subset (SR subset; *N* = 74), and patients who discontinued steroids were into steroid withdrawal subset (SW subset; *N* = 51).

The doses, start and end dates at the initiation, maximum and the final doses of oral corticosteroids were recorded. The final steroid dose was noted as the minimum dose before relapse or at the final visit. The intervals between the steroid initiation to the maximum doses, the steroid initiation to the stable status, and the drug reducing duration were calculated by month. The drug reducing duration (month) was defined as the interval from the end date of the maximum dose of oral steroids to the start date of the final dose. The accumulated dose of oral steroids before stable status was counted according to the medical record and periodic follow-up records. The average reduction speed (mg/month) was computed as the difference between the maximal dose and the final dose, divided by the drug reducing duration. Duration of the final dose was the interval from the start of the final dose to the last follow-up or relapse, whichever came first. In SW subset, the start date of the final dose was when the patient stopped the corticosteroid.

### Relapse

The primary endpoint was the occurrence of disease relapse, which was defined as recurrence of MG symptoms or a substantial increase in MG medications after the patient achieving MMS or better status (14). If patients didn't pay return visits during disease exacerbations, symptoms, and ADL-scores of relapses would be inquired by telephone. In this case, increases of ADL scores were determined as disease relapse. Patients of each subset were divided into relapse group (R-MG) and non-relapse group (NR-MG). Clinical features and therapeutic data were compared between R-MG and NR-MG groups to find relevant factors of relapse.

### Statistical Analysis

Continuous variables were presented as median and interquartile range (IQR), and categorical variables were presented as number and frequency. Clinical characteristics were compared between R-MG and NR-MG. Continuous data were analyzed using the Mann–Whitney U test. Categorical variables were analyzed using the chi-square or Fisher exact test, as appropriate. Candidate variables were included in Cox proportional hazards regression for calculating the multivariable hazard ratio (HR) if univariate *P*-values were < 0.10. Kaplan–Meier curves of relapse rates were plotted to illustrate the differences over time. Patients were censored at the last follow-up visits. The receiver operating characteristic (ROC) curve method was used to evaluate the best cut-off value of drug reducing duration in predicting relapse. Data were analyzed using SPSS (Version 22, IBM) and Prism (version7, GraphPad). A value of *P* lower than 0.05 was regarded as significant.

## Results

### The Primary Endpoint and the Therapy Regimens of All the Enrolled Patients

A relapse rate of 29.6% (37/125) was observed in the current study, and the median time from stable status to relapse was 18 months (IQR 8.0–21.5, range 2.0–53.0). Basic clinical characteristics and therapeutic data of the 125 patients are shown in Table 1. Ninety-seven patients had pure ocular symptoms at onset, six patients had pure limb weakness at onset, and seven patients had pure bulbar symptoms. Fifteen patients presented with more than one symptom at onset. No patient presented with shortness of breath at onset. The induction therapy regimens varied and could be generally categorized as steroid-increasing regimen, medium-dose regimen, and steroid-tapering regimen. Fifty-eight patients (46.4%) took low initial doses (median 15.0 mg/day, IQR 15.0–16.3) and the dosages gradually increased to maximal doses (median 35.0 mg/day, IQR 30.0–50.0) until improvement was observed; 45 patients (36.0%) took medium doses (median 25.0 mg/day, IQR 17.5–30.0) as maintenance therapy; and 22 patients (17.6%) initiated high dose corticosteroid treatments (median 60.0 mg/day, IQR 50.0–60.0), after which the dosages were gradually tapered. Other than drug reduction or discontinuation, the reported causes of relapse included over exertion (*N* = 4), cold (*N* = 3), and pneumonia (*N* = 1). Thirty-one patients experienced one MG relapse and six patients had two relapses during the follow-up periods.

**Table 1:** Clinical characteristics and therapeutic data of enrolled patients on steroid monotherapy.

	**Total (*N* = 125)**	**Steroid reduction subset (*****N*** **=** **74)**			**Steroid withdrawal subset (*****N*** **=** **51)**		
		**Relapse group**	**Non-relapse group**	***P*-value^**a**^**	**Relapse group**	**Non-relapse group**	***P*-value^**b**^**
		**(*N* = 19)**	**(*N* = 55)**		**(*N* = 18)**	**(*N* = 33)**	
Age at onset (years)	48.0 (IQR 35.0–59.0)	52.0 (IQR 42.0–63.0)	49.0 (IQR 34.0–57.5)	0.369	48.5 (IQR 36.5–59.0)	43.0 (IQR 28.5–62.0)	0.413
Sex (male)	73 (58.4%)	12 (63.2%)	29 (52.7%)	0.303	12 (66.7%)	20 (60.6%)	0.669
Symptoms at onset							
Ocular	111 (88.8%)	19 (100.0%)	47 (85.5%)	0.081	15 (83.3%)	30 (90.9%)	0.354
Limb	13 (10.4%)	1 (5.3%)	8 (14.5%)	0.267	0 (0.0%)	4 (12.1%)	0.164
Bulbar	17 (13.6%)	0 (0.0%)	9 (16.4%)	0.058	6 (33.3%)	2 (6.1%)	0.017
Symptoms at nadir							
Ocular	116 (92.8%)	19 (100.0%)	52 (94.5%)	0.405	15 (83.3%)	30 (90.9%)	0.354
Limb	34 (27.2%)	2 (10.5%)	18 (32.7%)	0.060	5 (27.8%)	9 (27.3%)	0.608
Bulbar	46 (36.8%)	6 (31.6%)	25 (45.5%)	0.291	8 (44.4%)	7 (21.2%)	0.082
Respiratory	8 (6.4%)	1 (5.3%)	5 (9.1%)	0.513	1 (5.6%)	1 (3.0%)	0.657
Disease severity at nadir				0.861			0.353
OMG (MGFA I)	64 (51.2%)	9 (47.4%)	24 (43.6%)		9 (50.0%)	22 (66.7%)	
Mild (MGFA II)	52 (41.6%)	9 (47.4%)	26 (47.3%)		7 (38.9%)	10 (30.3%)	
Moderate to severe (MGFA III-V)	9 (7.2)	1 (5.3%)	5 (9.1%)		2 (11.1%)	1 (3.0%)	
Autoimmune antibodies				0.323			0.097
AChR	90 (72.0%)	12 (63.2%)	42 (76.4%)		16 (88.9%)	20 (60.6%)	
MuSK	8 (6.4%)	3 (15.8%)	3 (5.5%)		0 (0.0%)	2 (6.1%)	
DN	27 (21.6%)	4 (21.2%)	10 (18.2%)		2 (11.1%)	11 (33.3%)	
RNS result	65 (52.0%)	9 (47.4%)	29 (52.7%)	0.445	9 (50.0%)	18 (54.4%)	0.756
Thymoma	17 (13.6%)	2 (10.5%)	10 (18.2%)	0.362	4 (22.2%)	1 (3.0%)	0.047
Thymectomy	33 (26.4%)	3 (15.8%)	17 (30.9%)	0.201	5 (27.8%)	6 (18.2%)	0.325
Presence of other autoimmune disease	6 (4.8%)	1 (5.3%)	4 (7.3%)	0.618	0 (0.0%)	1 (3.0%)	0.647
ADL score at nadir	4.0 (IQR 3.0–6.0)	4.0 (IQR 3.0–7.0)	4.0 (IQR 3.0–6.0)	0.844	5.0 (IQR 4.0–7.8)	4.0 (IQR 3.0–6.0)	0.254
Age at start of GC (years)	49.0 (IQR 35.5–59.5)	54.0 (IQR 44.0–63.0)	50.0 (IQR 36.0–59.0)	0.284	48.5 (IQR 36.5–59.0)	45.0 (IQR 31.5–62.0)	0.436
Disease course before immunotherapy (month)	5.0 (IQR 2.0–14.0)	6.0 (IQR 3.0–43.0)	5.0 (IQR 3.0–12.0)	0.434	4.0 (IQR 1.8–12.0)	2.0 (IQR 1.0–9.0)	0.445
Initial oral GC dose (mg/day)	20.0 (IQR 15.0–30.0)	15.0 (IQR 10.0–20.0)	20.0 (IQR 15.0–30.0)	0.242	20.0 (IQR 15.0–36.3)	20.0 (IQR 15.0–40.0)	0.772
Maximal oral GC dose (mg/day)	35.0 (IQR 25.0–50.0)	25.0 (IQR 20.0–50.0)	35.0 (IQR 25.0–50.0)	0.197	40.0 (IQR 28.8–60.0)	30.0 (IQR 20.0–47.5)	0.090
Final oral GC dose (mg/day)	5.0 (IQR 0.0–6.9)	5.0 (IQR 5.0–10.0)	5.0 (IQR 5.0–10.0)	0.445	0	0	-
Duration of the final dose (month)	6.0 (IQR 2.0–13.0)	3.0 (IQR 1.0–6.0)	5.0 (IQR 0.0–9.0)	0.396	4.0 (IQR 2.0–9.5)	17.0 (IQR 11.5–26.5)	<0.001
GC dose regimen of induction therapy				0.272			0.058
Steroid tapering regimen	22 (17.6%)	2 (10.5%)	8 (14.5%)		4 (22.2%)	8 (24.2%)	
Medium dose regimen	45 (36.0%)	9 (47.4%)	15 (27.3%)		4 (22.2%)	17 (51.5%)	
Steroid increasing regimen	58 (46.4%)	8 (42.1%)	32 (58.2%)		10 (55.6%)	8 (24.2%)	
Duration from GC initiation to stable condition (month)	3.0 (IQR 2.0–5.0)	2.0 (IQR 1.0–4.0)	3.0 (IQR 2.0–5.0)	0.090	3.0 (IQR 2.8–6.5)	2.0 (IQR 1.0–3.5)	0.024
Accumulated GC doses before stable status (mg)	2390.0 (IQR 1415.0–4495.0)	2055.0 (IQR 1350.0–3600.0)	3000.0 (IQR 1825.0–4950.0)	0.066	2727.5 (IQR 2185.0–6493.8)	1425.0 (IQR 900.0–2730.0)	0.009
Drug reducing duration (month)	13.0 (IQR 7.5–19.5)	8.0 (IQR 5.0–11.0)	15.0 (IQR 12.0–23.0)	<0.001	13.5 (IQR 3.0–20.75)	9.0 (IQR 3.5–16.0)	0.417
Average reduction speed(mg/month)	2.5 (IQR 1.4–5.0)	2.5 (IQR 1.4–6.3)	1.7 (IQR 1.0–3.5)	0.086	3.2 (2.2–13.8)	3.8 (1.9–8.8)	0.890
IVIG before remission	14 (11.2%)	1 (5.3%)	7 (12.7%)	0.337	3 (16.7%)	3 (9.1%)	0.354
IVMP before remission	7 (5.6%)	0 (0.0%)	4 (7.3%)	0.296	2 (11.1%)	1 (3.0%)	0.282

*^a^P-value was compared between R-MG and NR-MG in the steroid reduction subset; ^b^P-value was compared between R-MG and NR-MG in the steroid withdrawal subset; MGFA, Myasthenia Gravis Foundation of America classification; AChR, acetylcholine receptor; MuSK, muscle specific kinase; DN, double negative; ADL, Activities of Daily Living; GC, glucocorticoid; IVIG, intravenous immunoglobulin; IVMP, intravenous methylprednisolone; IQR, interquartile range*.

### Factors Correlative With Relapse During Steroid Reduction (SR)

Seventy-four patients were included in the SR subset, and 19 of them (25.7%) relapsed (Table 1). The median time from stable status to relapse was 16 months (IQR 7.0–19.0, range 2.0–31.0). Nine patients (12.2%) relapsed within the first year after achieving stable status and 17 patients (23.0%) relapsed within the first 2 years.

**Figure 2:**
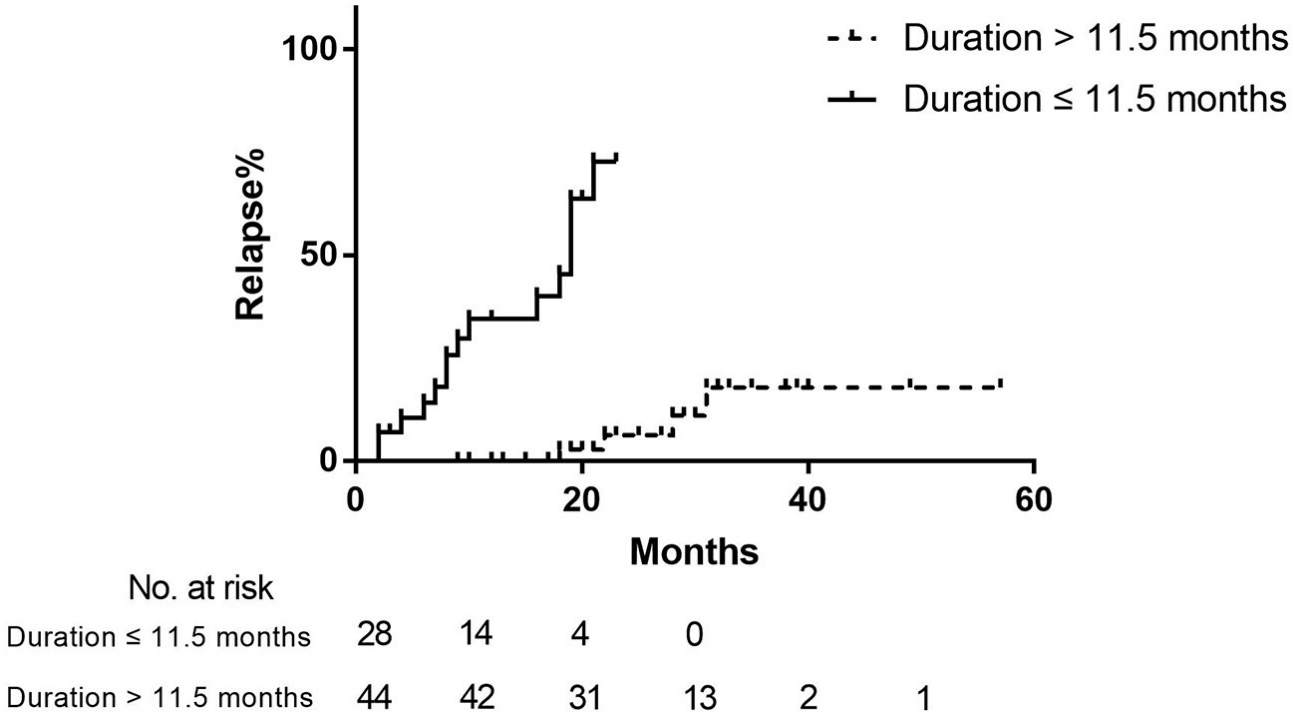
Kaplan–Meier curve of the relapse rate in SR patients with different steroid-reducing durations. Day 0 was defined as the date when patients achieved stable status.

Among clinical characteristics and therapeutic data between R-MG and NR-MG groups, the drug reducing duration was the only factor associated with relapse (R-MG median 8.0 months, IQR 5.0–11.0 vs. NR-MG median 15.0 months, IQR 12.0–23.0, *P* < 0.001). There were no statistical differences in sex, age at onset, onset symptom, disease severity at nadir, MG-ADL score at nadir, MG autoantibody, RNS result, thymoma, thymectomy or presence of other autoimmune diseases. Ages at the initiation of corticosteroid, disease courses before treatment, initial steroid doses, maximal doses, intervals from steroid initiation to stable condition and the number of patients taking IVIG or IVMP therapies were comparable between R-MG and NR-MG groups. The median steroid dose of patients at relapse was 5 mg/day (IQR 5.0–10.0), which was similar to that of NR-MG patients at last follow-ups (median 5 mg/day, IQR 5.0–10.0, *P* = 0.445). Patients in the R-MG group had shorter durations from steroid initiation to stable status (median 2.0 months, IQR 1.0–4.0 vs. NR-MG median 3.0 months, IQR 2.0-5.0; *P* = 0.090), lower accumulated doses of oral steroids before stable status (median 2055.0 mg, IQR 1350.0–3600.0 vs. NR-MG median 3000.0 mg, IQR 1825.0–4950.0; *P* = 0.066), and faster average reduction speeds of steroids (median 2.5 mg/month, IQR 1.4–6.3 vs. NR-MG median 1.7 mg/month, IQR 1.0–3.5; *P* = 0.086), which, however, didn't reach the statistical significance. In using the Cox proportional hazards model to identify the prognostic covariates associated with relapse during tapering steroid doses, day 0 was defined as the date when the patient achieved stable status. Only shorter drug reducing duration was identified as a significant predictor of relapse (HR = 0.81, 95%CI 0.74–0.89, *P* < 0.001; Table 2). Using the ROC curve, the best cut-off value of drug reducing duration (month) was 11.5 (sensitivity 74.5%, specificity 78.9%, area under the curve 0.779). Risk of relapse was augmented if the drug reducing duration was <11.5 months (HR 27.80, 95%CI 5.88–131.57, *P* < 0.001, Figure 2).

**Table 2:** Associations of clinical and therapeutic variables with relapse of patients on steroid reduction therapy (*N* = 74).

**Clinical and therapeutic variables**	**HR**	**95% CI**	***P*-value**
**Univariate**			
Drug reducing duration (month)	0.81	0.74–0.89	<0.001
Drug reducing duration <11.5 months	27.80	5.88–131.57	<0.001
Ocular weakness at onset	24.22	0.05–12422.17	0.317
Bulbar weakness at onset	0.04	0.00–17.62	0.301
Limb weakness at nadir	3.37	0.78–14.58	0.105
Duration from GC initiation to stable condition (month)	0.90	0.71–1.12	0.339
Accumulated GC doses before stable status (mg)	1.00	1.00–1.00	0.570
Average reduction speed(mg/month)	1.02	0.97–1.07	0.422
Thymectomy	1.87	0.54–6.44	0.321

*Variables were included in multivariate analyses if P < 0.10 in univariate analyses; GC, glucocorticoid; HR, hazard ratio*.

### Factors Correlative With Relapse After Steroid Withdrawal (SW)

**Figure 3:**
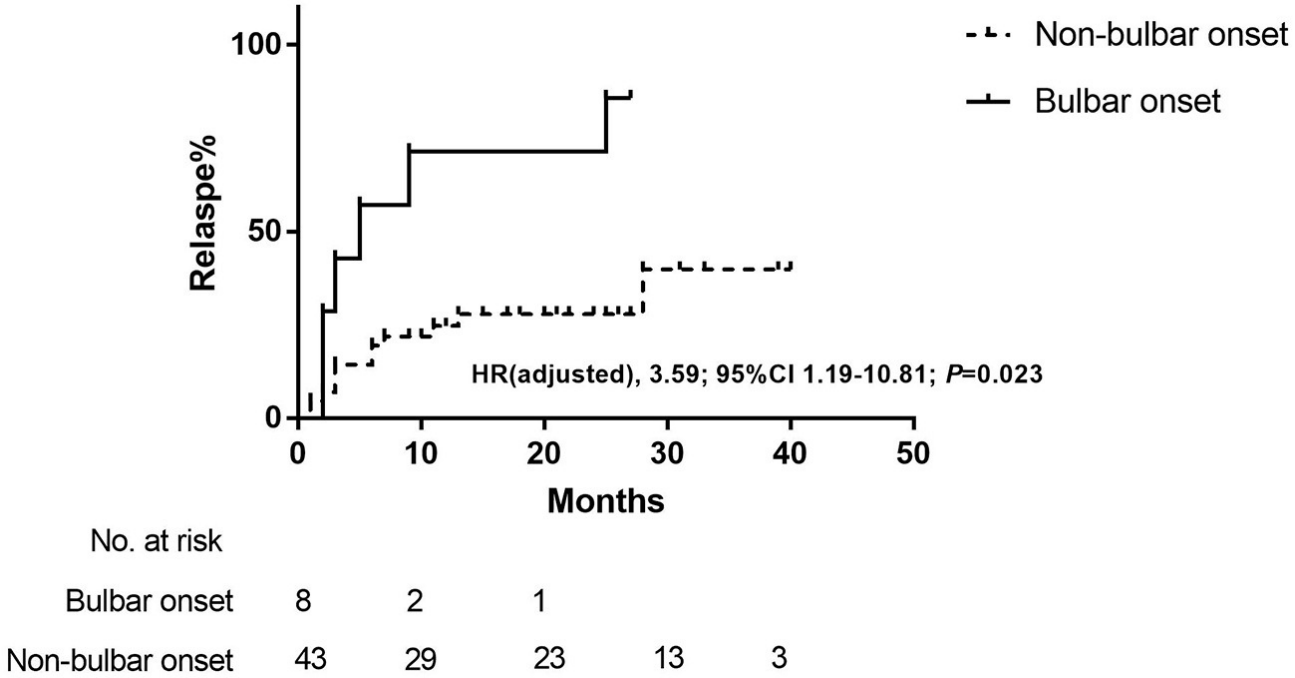
Kaplan–Meier curve of the relapse rate after steroid discontinuation in patients with bulbar onset and non-bulbar onset. Day 0 was defined as the steroid discontinuation date. HR indicated hazard ratio.

Fifty-one patients were included in the SW subset (Table 1). The occurrence of CSR was observed in 25/125 (20.0%) enrolled MG patients. Eighteen (35.3%) patients experienced relapses after drug discontinuation, and 15 of them relapsed within 12 months. The median time from stable status to relapse was 20 months (IQR 8.0–23.5, range 3.0–53.0). The median time from steroid withdrawal to relapse was 4 months (IQR 2.0–9.5, range 1.0–40.0). In patients who discontinued the steroids, relapse was associated with the onset symptom of bulbar weakness (*P* = 0.017; odds ratio: 7.75, 95%CI 1.37–43.87), thymoma (*P* = 0.047; odds ratio: 9.14, 95%CI 0.94–89.35), duration from steroid initiation to stable status (R-MG median 3.0 months, IQR 2.8–6.5 vs. NR-MG median 2.0 months, IQR 1.0–3.5; *P* = 0.024) and accumulated steroid doses before stable status (R-MG median 2727.5 mg, IQR 2185.0–6493.8 vs. NR-MG median 1425.0, IQR 900.0–2730.0; *P*=0.009). In Cox proportional hazards regression analysis, day 0 was defined as the steroid discontinuation date. Bulbar weakness at onset was identified to have a significant association with relapses after steroid discontinuation (adjusted HR 3.59, 95%CI 1.19–10.81, *P* = 0.023; Table 3, Figure 3). The median time from steroid discontinuation to relapse for patients with bulbar onset was 4.0 months (IQR 2.0–21.0, range 0.0–27.0).

**Table 3:** Associations of clinical and therapeutic variables with relapse after steroid discontinuation (*N* = 51).

**Clinical and therapeutic variables**	**HR**	**95% CI**	***P* value**
**Univariate**			
Bulbar weakness at onset	4.34	1.59–11.85	0.004
Bulbar weakness at nadir	2.36	0.93–6.00	0.071
Thymoma	3.06	0.99–9.41	0.051
Thymectomy	0.62	0.22–1.76	0.369
Autoimmune antibodies			
AChR	Ref	-	0.309
MuSK	0.00	-	0.985
DN	0.32	0.07–1.38	0.125
GC dose regimen of induction therapy			
Steroid tapering regimen	Ref.	-	0.197
Medium dose regimen	0.40	0.10–1.62	0.200
Steroid increasing regimen	1.15	0.36–3.73	0.813
Maximal oral GC dose (mg/day)	1.02	0.99–1.04	0.209
Duration from GC initiation to stable status (month)	1.05	0.99–1.12	0.131
Accumulated GC doses before stable status (mg)	1.00	1.00–1.00	0.132
**Multivariate**			
Bulbar weakness at onset	3.59	1.19–10.81	0.023
Thymoma	1.82	0.53–6.28	0.342
**Multivariate**			
Bulbar weakness at nadir	2.20	0.86–5.67	0.101
Thymoma	2.77	0.89–8.64	0.079

*Variables were included in multivariate analyses if P < 0.10 in univariate analyses; AChR, acetylcholine receptor; MuSK, muscle specific kinase; DN, double negative; GC, glucocorticoid; HR, hazard ratio*.

## Discussion

We demonstrated a relapse rate of 29.6% in a cohort of well-managed MG patients taking GC as monotherapy and 20.0% of enrolled patients achieved CSR by the end of the study, which was consistent with previous studies (5, 7–10). Analysis implied that shortened GC reducing duration was a significant predictor for relapse during steroid tapering in the well-controlled MG patients. The bulbar symptom at disease onset was independently associated with relapse after the discontinuation of GC.

Among patients in SR subset of the current study, 25.7% patients relapsed. Most relapses (17/19, 89.5%) happened within the first 2 years after achieving stable status. R-MG group had significantly shorter drug reducing duration than NR-MG, and there were numerical trends of less duration from steroid initiation to stable status, lower accumulated steroid doses before stable status and higher steroid-reducing speed in R-MG group, which were approaching significance, whereas no statistical difference was found in clinical characteristics. Moreover, shortness of steroid-reducing duration was identified to be associated with increased risk of relapse during steroid tapering by the Cox proportional hazards model. These results implied that relapses during steroid reduction were more relevant to inadequate treatments. It was validated that once generalized MG patients attained the MMS, depending on the efficacy of azathioprine, rapid tapering of prednisone was associated with good outcomes and well tolerated without destabilizing MG (15). However, when the steroid was administered in absence of other immunosuppressants, it was well acknowledged that rapid dose-reduction could result in a recurrence of weakness (5, 11, 12). In line with these data, we found that risk of relapse increased by 26-fold when the steroid-reducing durations of the patients were <11.5 months. The findings led us to conclude that the steroid-reducing duration of at least 1 year might be in favor of preventing disease relapse. It was close to statistically significant that a relatively high average reduction speed was observed in R-MG groups. The result was in accordance with less drug reducing duration in R-MG and might reach significance when expanding the sample size. The final doses before relapses in our study were similar to the minimum doses in the NR-MG groups, which was 5 mg/day (IQR 5.0–10.0). Low-dose medication could preserve well management of MG (11, 16). The side effects resulting from long-term use of steroids were dose-dependent, which could be minimized and acceptable by administration of dosages no more than 5 mg (17, 18). In Japanese guidelines for MG, MM with oral prednisolone (PSL) of 5 mg/day or below was recommended as the therapeutic goal (19), which was more reachable than CSR and with equivalent satisfaction of patients (20). Since only shorter drug reducing duration was identified as a significant predictor of relapse, we presumed that with steroid-reducing duration longer than 1 year, patients might maintain asymptomatic on oral steroids of 5 mg per day.

Among patients who stopped GC therapies, 35.3% patients relapsed and most relapses (15/18, 83.3%) happened within the first year after GC discontinuation. To our knowledge, this is the first report concerning prognostic factors of relapse after steroid discontinuation. In the present study, bulbar weakness at onset was identified as a predictor of relapse in patients who discontinued steroids. Manifestations of bulbar symptoms included dysarthria, dysphagia, and dysphonia (21, 22), which might be the initial and solitary presentation in 15–27% MG patients (23, 24). The bulbar symptom was reported to be one of risk factors of the postsurgery myasthenia crisis (25). The relationship between the onset phenotype involving bulbar muscles and elevated relapse risk had not yet been published yet. Presence of thymoma and severe forms of MG were identified as risk factors of relapse in a cohort of steroid-treated MG patients (6). However, it would be more reasonable if they had performed a multivariate analysis and considered the confounding factors. It was demonstrated that patients with thymoma were generally in serious conditions (26). In agreement with pervious study, we observed a significantly higher proportion of patients with thymoma in R-MG, whereas it did not achieve statistical significance in the Cox regression analysis. However, it should be taken into account that our study was limited by the small sample size. In the present study, durations of oral steroid and accumulated GC doses before the stable status were significantly higher in R-MG of the SW subset, indicating that severe diseases might be related to relapses. Nevertheless, there were no differences in ADL scores or disease severity at nadir. This can be explained by the fact that patients in the current study were generally with mild to moderate diseases, since the median ADL score at maximal worsening of the cohort was 4 points. Patients with severe forms might take combining non-steroidal immunotherapies (8, 11) and were excluded from the current study. Taken together, bulbar weakness at onset could be indicative of relapse after GC discontinuation and patients might require long-term use of steroids or other immunosuppressants.

Our findings could not be ascribed to the confounding effects of MG antibodies or thymectomy, as the autoantibodies and thymectomized patients did not statistically significantly differ between R-MG group and NR-MG group. Our results coincided with the previous report that no significant correlation was found between thymectomy and relapse (7). Even though thymectomy was validated in controlling diseases and sparing prednisone doses in non-thymomatous generalized MG patients (27), relapse remained a major concern after discontinuing pharmacotherapy in thymectomized patients (28). This might be attributed to the fact that the disease relevant lymphocytes shuttled from thymus into circulation and resided in secondary sites of chronic pathogenic antibody production (29, 30). Thus, precautions should be taken against disease relapses when thymectomized patients become symptom-free and discontinue the immunotherapies. MuSK-MG was demonstrated to be associated with a higher risk of relapse (8). However, when comparing our results to the previous study, it must be pointed out that the majority of MuSK-MG patients in our cohort were ascribed to other immunotherapies and were excluded from the current study.

The main limitation of our work was the retrospective design and the limited sample size from a single center. Besides, follow-ups were completed mainly by telephones after patients achieved stable status. Therefore, the maintenance of stable conditions was based on the self-reports of patients, instead of careful physical examinations. Because of the retrospective design, patients who switched to other immunotherapies due to steroid-induced side effects during the follow-up periods were excluded from the study. Therefore, steroid maintenance therapies were well tolerated in the current cohort and the side effects of steroid were not measured and compared between groups.

In conclusion, despite the satisfactory effects of corticosteroids, about 30% well-managed patients with MG might experience disease relapses. Our study emphasized the significance in prolonged steroid-reducing durations of at least 1 year before reaching maintenance doses to prevent relapses. Moreover, laryngological manifestations at the onset of a disease might predict a high risk of relapse after discontinuance of GC, and these patients should be proposed to take long-term steroids or other immunosuppressants.

## Data Availability Statement

The raw data supporting the conclusions of this article will be made available by the authors, without undue reservation.

## Ethics Statement

The studies involving human participants were reviewed and approved by Ethics Committee of Xuanwu Hospital, Capital Medical University, China (No. 2017084). The patients/participants provided their written informed consent to participate in this study.

## Author Contributions

SS contributed to this work in study design, collecting data, statistical analysis, drafting, and revising the manuscript. LL, ZF, SZ, QW, JW, YL, LD, MW, and HC contributed with the enrollment of patients and acquisition of data. YD contributed with study concept and design, drafting, revising the manuscript, and interpretation of the data. All authors contributed to the article and approved the submitted version.

## Funding

This work was supported by the Clinical Cohort Study of Myasthenia Gravis, National Key R&D Program of China, Precision Medicine Project (No. 2017YFC0907700), and the National Natural Science Foundation of China (No. 62171299).

## Conflict of Interest

The authors declare that the research was conducted in the absence of any commercial or financial relationships that could be construed as a potential conflict of interest.

## Publisher's Note

All claims expressed in this article are solely those of the authors and do not necessarily represent those of their affiliated organizations, or those of the publisher, the editors and the reviewers. Any product that may be evaluated in this article, or claim that may be made by its manufacturer, is not guaranteed or endorsed by the publisher.
